# Pelvic floor muscle exercises alleviate symptoms and improve mental health and rectal function in patients with low anterior resection syndrome

**DOI:** 10.3389/fonc.2023.1168807

**Published:** 2023-04-20

**Authors:** Wu Yuanyuan, He Shiyin, He Lei, Ding Ding

**Affiliations:** Department of General Surgery, The Third Affiliated Hospital of Anhui Medical University (The First People's Hospital of Hefei), Hefei, Anhui, China

**Keywords:** pelvic floor muscle exercises, rectal function, low anterior resection syndrome, fecal incontinence, rectal cancer

## Abstract

**Background:**

Pelvic floor rehabilitation has been reported to be effective in improving fecal incontinence. The aim of this study was to prospectively evaluate the effectiveness of combined pelvic floor muscle exercises (PFMEs) and loperamide treatment on rectal function and mental health for low anterior resection syndrome (LARS) patients after sphincter-saving operation (SSO) for rectal cancer.

**Methods:**

A total of 60 inpatients diagnosed with LARS were enrolled and randomly assigned to one of two groups: patients in Group A (n = 30) were treated with a PFME intervention and those in Group B (n = 30) with a control intervention for 4 weeks. High-resolution anorectal manometry (HRAM) was performed for all LARS patients. Demographic information was collected for all patients, and they subsequently also completed several questionnaires, including the Hospital Anxiety and Depression Scale (HADS), a measure of Wexner score, a measure of stool frequency per day, and the Bristol Stool Form Scale (BSFS).

**Results:**

No significant differences between the groups were observed in baseline data. With regard to rectal function, we found significant improvements at week 4 in maximal resting pressure (MRP) (39.93 ± 5.02 *vs.* 28.70 ± 5.40 mmH_2_O, p < 0.001) and maximal squeeze pressure (MSP) (132.43 ± 8.16 mmH_2_O *vs.* 113.33 ± 9.87 mmH_2_O, p < 0.001) among Group A patients compared to Group B patients. Additionally, Wexner scores were significantly lower in Group A than in Group B at week 4 (8.10 ± 1.24 *vs.* 9.87 ± 1.29 ml, p = 0.018), as were stool frequency (6.47 ± 0.90 *vs.* 7.83 ± 0.93, p < 0.001) and BSFS scores (5.17 ± 0.65 *vs.* 6.10 ± 0.80, p = 0.020). Notably, HADS scores were also significantly lower in Group A than in Group B at week 4 (8.25 ± 2.36 *vs.* 10.48 ± 3.01, p < 0.001). Additionally, both anxiety scores (4.16 ± 1.38 *vs.* 5.33 ± 1.69, p < 0.001) and depression scores (4.09 ± 1.56 *vs.* 5.15 ± 1.89, p < 0.001) were significantly lower in Group A than in Group B at week 4.

**Conclusion:**

Pelvic floor muscle exercises are an effective treatment that can alleviate symptoms and improve rectal function and mental health in patients with low anterior resection syndrome.

Total mesorectal excision (TME) is recognized as an important technique in treating rectal cancer (1). Carrying out TME can provide the best oncologic results because the entire tumor is removed (2). A sphincter-saving operation (SSO) is generally considered to be more humanistic than permanent colostomy (PC) with regard to quality of life because it avoids the need for a permanent stoma. SSO is implemented when the rectal cancer is located near the anal sphincter complex, and more than 50% of such patients can successfully avoid a permanent colostomy (3). In addition to survival-related outcomes, such as overall survival (OS) and failure-free survival (FFS) rates, functional outcomes have gradually become valued to an increasing extent and have become a vital index of the success of a procedure (4).

However, low anterior resection syndrome (LARS) usually occurs as a potential consequence of SSO (5). Bowel function can be impaired by low anorectal mobilization and rectal excision, and LARS consists of any altered defecation status, including emptying difficulties, incomplete evacuation, urgency, and fecal incontinence. Short-term LARS, defined as symptoms that resolve within 6–12 months after SSO, is most likely to be attributable to short-lived neorectal irritability in the postoperative period, while long-term LARS, defined as symptoms continuing for more than 12 months after surgery, are usually due to permanent changes (6, 7). A previous study has reported a high prevalence of LARS, with more than 80% of patients who undergo SSO experiencing the syndrome with varying degrees of severity (8).

There are limited treatment options that can alter the natural course of LARS. To date, a small number of strategies have been proven effective for LARS symptoms, including dietary modification, the use of incontinence pads, and pharmacotherapy (5). Therefore, it is necessary to investigate the efficacy of complementary and alternative medicine (CAM) for LARS. Pelvic floor rehabilitation has been reported to be effective in improving fecal incontinence (9, 10). The aim of this study was to prospectively evaluate the effectiveness of combined pelvic floor muscle exercises (PFMEs) and loperamide treatment on rectal function and mental health for LARS patients after SSO for rectal cancer.

## Patients and methods

### Patients

A total of 63 patients were eligible to participate in this research. However, three patients were excluded from the study due to their inability to keep exercising. The remaining 60 inpatients (ages 18–75) who underwent SSO for rectal cancer were enrolled. Patients were recruited to participate in this study at the Third Affiliated Hospital of Anhui Medical University between October 2019 and October 2022. The primary symptoms of LARS were defined as fecal incontinence, urgent evacuation, and frequent defecation. This was a pilot clinical experiment. The 60 participating patients with LARS were randomly divided into two groups: Group A (30 LARS patients) received the PFME intervention in addition to loperamide (2 mg, t.i.d.), and Group B (control group, 30 LARS patients) received loperamide (2 mg, t.i.d.) only.

The inclusion criteria were as follows: 1) a minimum LARS score of 21/42 (= at least minor LARS) (11) at 1 month after surgery (no ileostomy) or within 6 months after ileostomy closure; 2) no diverting stoma or reversal of temporary stoma at the time of participation in pelvic floor muscle exercises; and 3) tumor-free status as confirmed by pathological findings, endoscopic reports, abdominal ultrasound, and/or computed tomography data. The exclusion criteria were: 1) age > 75 years; 2) inability to continue exercising or discontinuing participation mid-program; and 3) temporary diverting ileostomy in place. The study protocol was approved by the Ethics Committee of the Third Affiliated Hospital of Anhui Medical University (No. 2022-75) and was registered on the Chinese Clinical Trial Registry (No. ChiCTR2300067873). Written informed consent was obtained from all participants before their inclusion in the study.

### Experimental protocol

The current study consisted of a randomized control trial in which participants were not blinded to their treatment assignment. Eligible patients were randomly divided into two groups: Group A (30 LARS patients) received the PFME intervention in addition to loperamide (2 mg, t.i.d.), and Group B (control group, 30 LARS patients) received only loperamide (2 mg, t.i.d.). Randomization was performed using Randomizer.org (https://www.randomizer.org/) with a 1:1 allocation ratio. All participants underwent high-resolution anorectal manometry and completed the Cleveland Clinic Florida Fecal Incontinence Score (Wexner score) and the Bristol Stool Form Scale (BSFS). All these outcomes were evaluated for all patients before and after the 4-week intervention period.

### High-resolution anorectal manometry

High-resolution anorectal manometry (HRAM) was performed for all LARS patients as described in a previous study (12). In brief, one dose of glycerin enema was administered for bowel preparation 30–60 min before the HRAM examination. A 12-channel anorectal manometric catheter was employed to measure maximal resting pressure (MRP) and maximal squeeze pressure (MSP). A computerized system was used to record and analyze the traces (MMS Database 8.6; Solar GI, Enschede, The Netherlands). HRAM was conducted before and after the final PFME session.

### Pelvic floor muscle exercise therapy

Patients in Group A were offered PFME therapy with duration of 4 weeks and evaluated via a manometry system. The amplitude and duration of the voluntary anal sphincter contractions were measured by a water-filled manometric anal probe, which was attached to a dedicated computer (ASE2000 Biofeedback Equipment; King Medicine, Changzhou, China). In addition, the PFME therapy included use of two different methods (13): strength training and sensory training. During a series of 10-s trials, repeated for 30 min during the intervention, patients were required to relax, squeeze, and strain (9). For strength training, patients were encouraged to contract their external anal sphincter without a balloon for 30 min twice daily. Sensory training was performed once daily using a rectal balloon distended at 5-ml intervals until the participant indicated the sensation. Subsequently, the volume of the balloon was increased in progressive 5-ml increments, and the patient was taught to contract their sphincter.

### Outcome measures

Patients were required to complete the Cleveland Clinic Florida Fecal Incontinence Score (Wexner score), ranging from 0 (perfect continence) to 20 (severe incontinence) (14). Additionally, the stool frequency per day of each patient was recorded. Patients were also evaluated on the BSFS, a validated 7-point assessment that ranges from 1 [indicating separate, hard lumps, like nuts (hard to pass)] to 7 [indicating watery, no solid pieces (entirely liquid)]. All these outcomes were evaluated for all patients before and after the 4-week intervention period (15). The Hospital Anxiety and Depression Scale (HADS), developed by Zigmond and Snaith (16) in 1983, was also administered. This instrument is mainly used to screen anxiety and depression symptoms in patients. It is composed of 14 items, seven of which are used to evaluate depression and seven to assess anxiety. There are three categories according to subscale score: no anxiety or depressive symptoms, suspected anxiety or depressive symptoms (8 to 10 points), and anxiety or depressive symptoms definitely present (11 to 21 points).

### Statistical analysis

SPSS software v.22.0 (IBM Corp., Armonk, NY, USA) was used for statistical analyses. For continuous variables, normally distributed data are presented in the form mean ± standard deviation, while non-normally distributed data were expressed as medians with the corresponding interquartile range. The two groups were compared using unpaired *t*-tests for normally distributed continuous data, and using the Mann–Whitney U-test for non-parametric data. The chi-square test was employed for categorical variables. p < 0.05 signified statistical significance.

## Results

### Clinical characteristics

**Figure 1:**
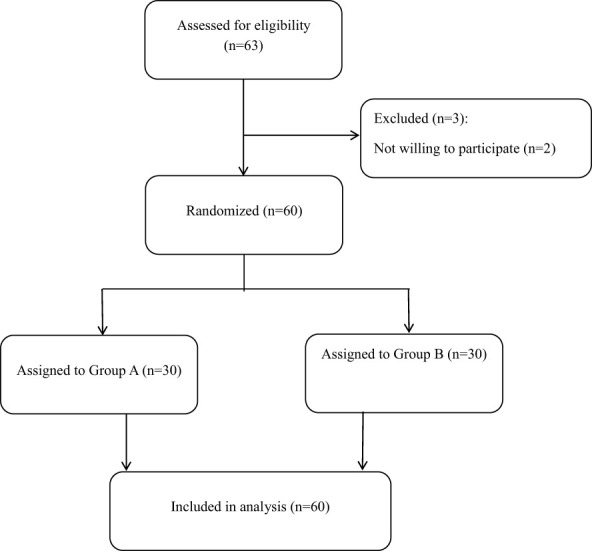
Flow chart of the study.

A total of 60 LARS patients were enrolled in this study and randomly assigned to one of two groups: the PFME intervention group (Group A) or the control group (Group B). A flow chart of participants is shown in **Figure 1**. In Group A, patients received PFME therapy in addition to loperamide (2 mg, t.i.d.) for a treatment duration of 4 weeks, while patients in the control group received only loperamide (2 mg, t.i.d.). Of the 60 LARS patients who completed the study, 22 were female and 38 were male, with a mean age of 61 (54, 68) years. No statistical differences in age, age at surgery, gender, body mass index (BMI), or distance of anastomosis from the anal verge were observed among the three groups (all p > 0.05). With regard to primary symptoms, 40 patients had fecal incontinence, 11 had urgent evacuation, and 9 had frequent defecation as the primary symptom. In addition, 23 LARS patients were at stages T1+T2 and 37 were at T3+T4. Notably, there were 11 patients (36.67%) with coloanal anastomosis in Group A and 10 (33.33%) in Group B. No statistical differences between the groups were noted in terms of primary symptom, cancer stage, number of laparoscopic surgeries, radiation therapy, chemotherapy, or diabetes (all p > 0.05). These results are shown in **Table 1**.

**Table 1:** Clinical characteristics of the two study groups (all patients with LARS).

	Overall (N = 60)	Group A (n = 30)	Group B (n = 30)	t/χ^2^	p
Age (years)	60.47 ± 6.62	59.93 ± 6.91	61.00 ± 6.38	0.621	0.537
Age at surgery (years)	56.73 ± 6.16	56.10 ± 6.97	57.37 ± 6.28	0.739	0.463
Gender (n)					
Male	38	20	18	0.278	0.592
Female	22	10	12		
BMI (kg/m^2^)	22.60 ± 3.71	22.67 ± 3.56	22.52 ± 3.92	0.148	0.883
Primary symptom (n)					
Fecal incontinence	40	21	19	0.302	0.860
Urgent evacuation	11	5	6		
Frequent defecation	9	4	5		
Distance of anastomosis from anal verge (cm)	4.809 ± 0.94	4.69 ± 0.99	4.91 ± 0.90	0.888	0.378
Coloanal anastomosis (n)	21	11	10	0.073	0.787
Laparoscopic surgery (n)	40	21	19	0.300	0.584
T stage (n)					
T1+T2	23	12	11	0.071	0.791
T3+T4	37	18	19		
Radiation therapy (n)	18	10	8	0.317	0.573
Chemotherapy (n)	28	15	13	0.268	0.605
Diabetes (n)	21	11	10	0.073	0.787

Data are presented as mean ± standard deviation or n. The difference between the two groups was analyzed using t-test or chi-square test.

LARS, low anterior resection syndrome; BMI, body mass index.

### Baseline data

Regarding HRAM parameters, we found no significant differences between the groups in MRP (28.70 ± 5.57 *vs.* 29.67 ± 5.77 mmH_2_O, p = 0.512) or MSP (112.83 ± 9.77 *vs.* 110.87 ± 8.39 mmH_2_O, p = 0.406). Regarding fecal incontinence symptoms, no difference in Wexner score was observed between patients in Group A and those in Group B (10.10 ± 1.35 *vs.* 10.30 ± 1.42, p = 0.578). There was also no significant difference between Groups A and B in stool frequency (8.50 ± 1.48 *vs.* 8.30 ± 1.44, p = 0.598) or BSFS score (6.20 ± 0.76 *vs.* 6.13 ± 0.78, p = 0.738). Additionally, there was no significant difference between Groups A and B in terms of total HADS score (11.32 ± 3.60 *vs.* 12.03 ± 4.06, p = 0.314). Thus, these parameters were comparable between the patients in the two groups. These results are shown in **Table 2**.

**Table 2:** Baseline data for the two study groups (all patients with LARS).

	Group A (n = 30)	Group B (n = 30)	t/χ^2^	p
MRP (mmH_2_O)	28.70 ± 5.57	29.67 ± 5.77	0.660	0.512
MSP (mmH_2_O)	112.83 ± 9.77	110.87 ± 8.39	0.837	0.406
Wexner score	10.10 ± 1.35	10.30 ± 1.42	0.560	0.578
Stool frequency (per day)	8.50 ± 1.48	8.30 ± 1.44	0.530	0.598
BSFS score	6.20 ± 0.76	6.13 ± 0.78	0.336	0.738
HADS score	11.32 ± 3.60	12.03 ± 4.06	0.879	0.314
Anxiety score	5.36 ± 1.59	5.68 ± 1.13	0.618	0.540
Depression score	6.34 ± 1.28	5.89 ± 1.94	0.651	0.512

Data presented as mean ± standard deviation.

LARS, low anterior resection syndrome; MRP, maximal resting pressure; MSP, maximal squeeze pressure.

### Effects of PFMEs on HRAM parameters

**Figure 2:**
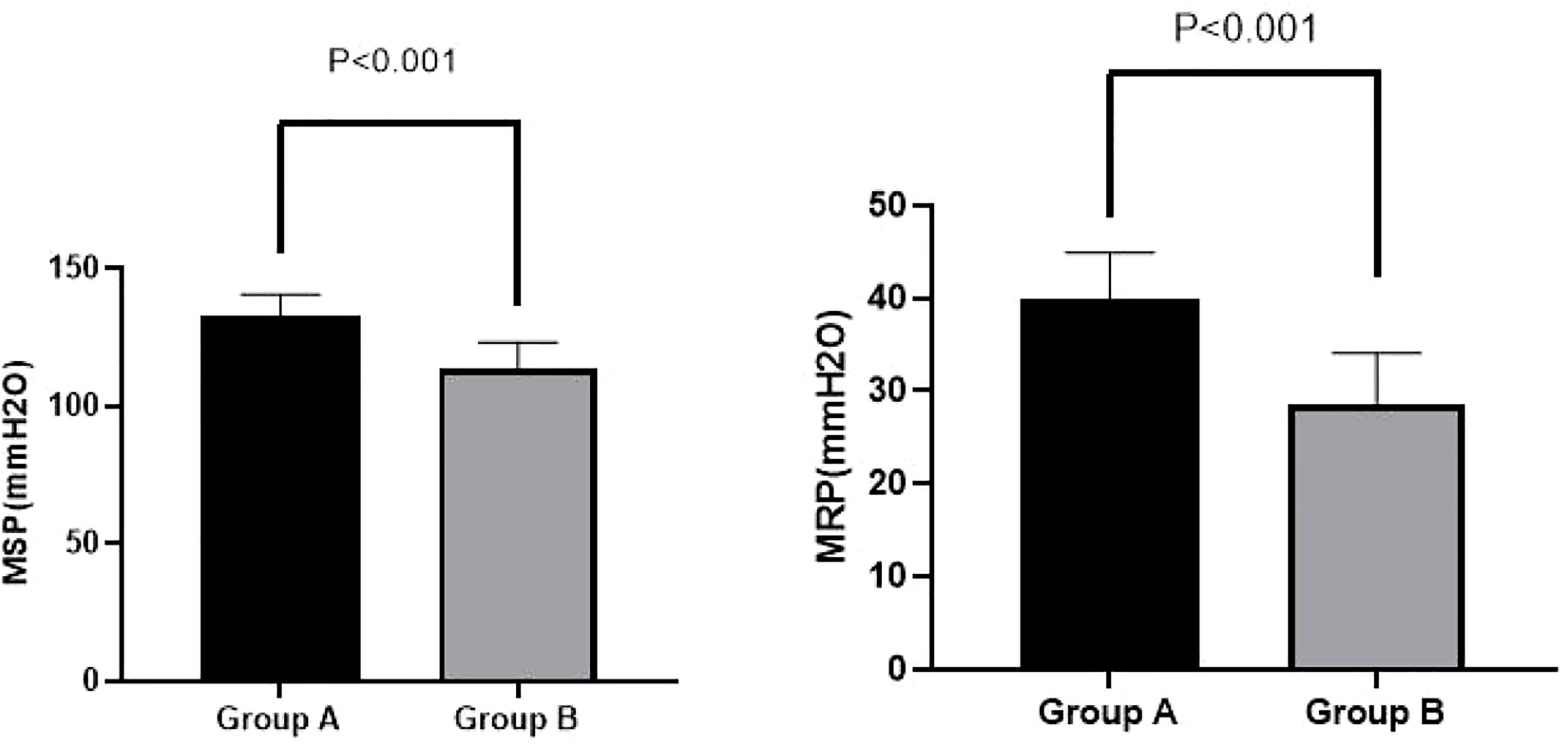
MSP and MRP in Groups A and B at week 4. A significant improvement in MRP (39.93 ± 5.02 *vs.* 28.70 ± 5.40 mmH_2_O, p < 0.001) and MSP (132.43 ± 8.16 *vs.* 113.33 ± 9.87 mmH_2_O, p < 0.001) for Group A compared with Group B was observed at week 4. MSP, maximal squeeze pressure; MRP, maximal resting pressure.

Interestingly, we found significant improvements at week 4 among Group A patients compared with Group B patients on MRP (39.93 ± 5.02 *vs.* 28.70 ± 5.40 mmH_2_O, p < 0.001) and MSP (132.43 ± 8.16 *vs.* 113.33 ± 9.87 mmH_2_O, p < 0.001). These results are shown in **Figure 2**.

### Effects of PFMEs on fecal incontinence symptoms

**Figure 3:**
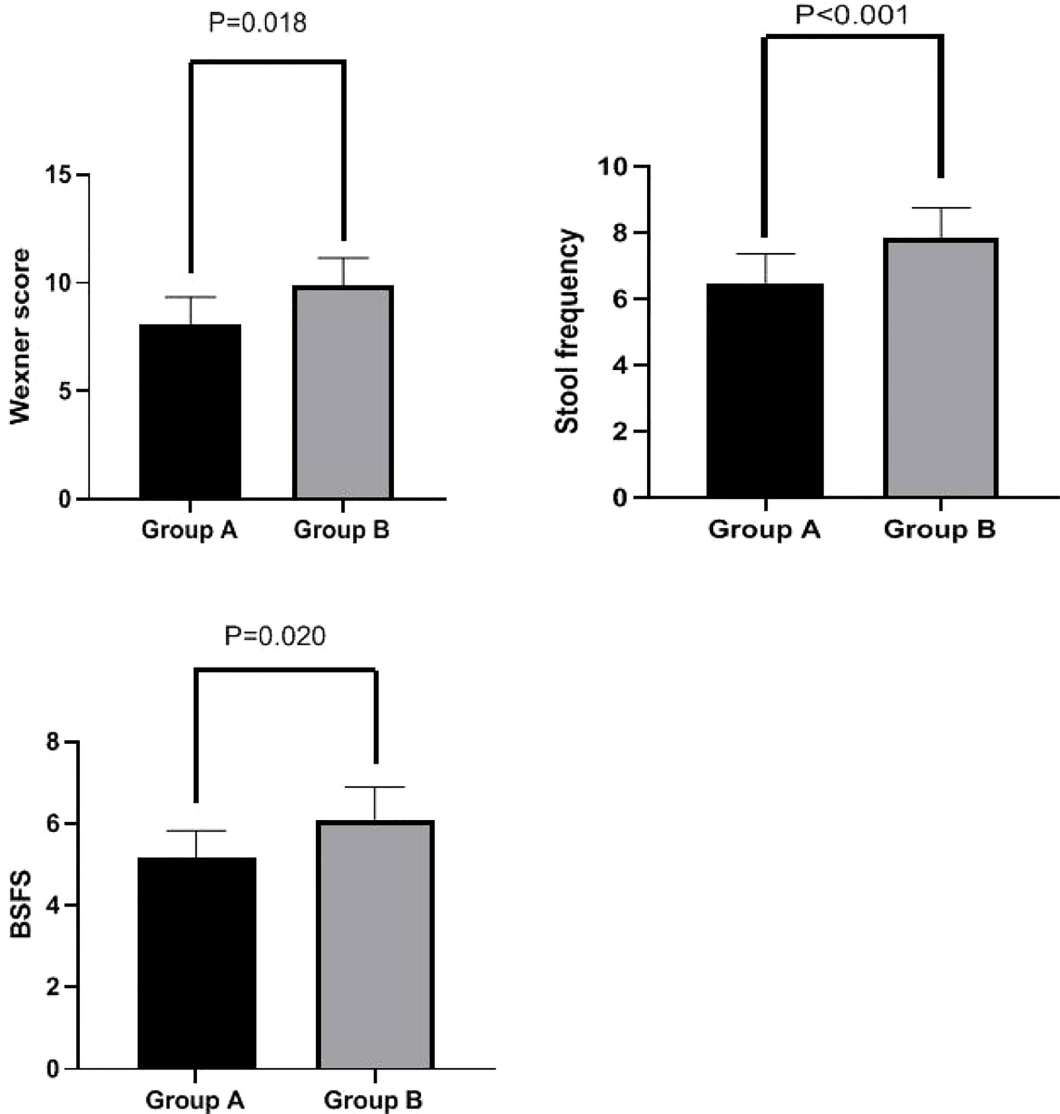
Wexner score, stool frequency, and BSFS score in Groups A and B at week 4. Wexner scores were significantly lower in Group A than in Group B at week 4 (8.10 ± 1.24 *vs.* 9.87 ± 1.29 ml, p = 0.018). Stool frequency (6.47 ± 0.90 *vs.* 7.83 ± 0.93, p < 0.001) and BSFS scores (5.17 ± 0.65 *vs.* 6.10 ± 0.80, p = 0.020) were also significantly lower in Group A than in Group B at week 4. BSFS, Bristol Stool Form Scale.

Wexner scores were significantly lower in Group A than in Group B at week 4 (8.10 ± 1.24 *vs.* 9.87 ± 1.29 ml, p = 0.018). Additionally, stool frequency (6.47 ± 0.90 *vs.* 7.83 ± 0.93, p < 0.001) and BSFS scores (5.17 ± 0.65 *vs.* 6.10 ± 0.80, p = 0.020) were significantly lower in Group A than in Group B at week 4. These results are shown in **Figure 3**.

### Effects of PFMEs on mental health

**Figure 4:**
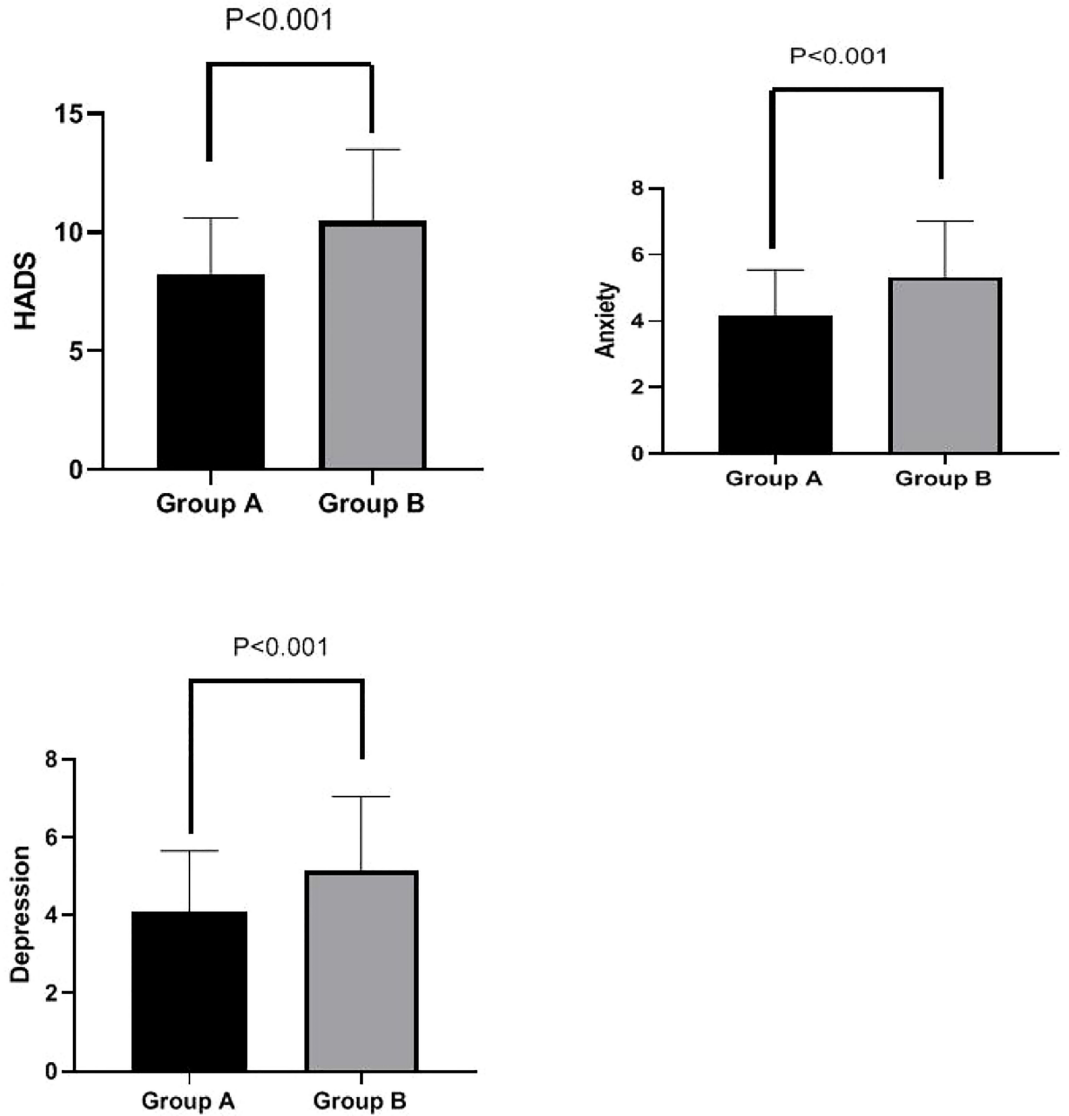
HADS total score, anxiety subscore, and depression subscore in Groups A and B at week 4. Total HADS scores were significantly lower in Group A than in Group B at week 4 (8.25 ± 2.36 *vs.* 10.48 ± 3.01, p < 0.001). Furthermore, anxiety scores (4.16 ± 1.38 *vs.* 5.33 ± 1.69, p < 0.001) and depression scores (4.09 ± 1.56 *vs.* 5.15 ± 1.89, p < 0.001) were both significantly lower in Group A than in Group B at week 4. HADS, Hospital Anxiety and Depression Scale.

HADS scores were significantly lower in Group A than in Group B at week 4 (8.25 ± 2.36 *vs.* 10.48 ± 3.01, p < 0.001). Additionally, both anxiety scores (4.16 ± 1.38 *vs.* 5.33 ± 1.69, p < 0.001) and depression scores (4.09 ± 1.56 *vs.* 5.15 ± 1.89, p < 0.001) were significantly lower in Group A than in Group B at week 4. These results are shown in **Figure 4**.

## Discussion

In the present study, we observed that a 4-week intervention involving pelvic floor muscle exercises resulted in improved external anal sphincter contraction in LARS patients. Furthermore, LARS patients receiving the PFME intervention reported significant improvements in stool frequency, symptoms of fecal incontinence (Wexner score), stool consistency, and mental health compared with the control group. Thus, participation in PFMEs resulted in improvements on subjective and objective measures for LARS patients at week 4 compared with the control group. Therefore, this study provides potential evidence that pelvic floor muscle exercises are an effective treatment and can alleviate symptoms and improve rectal function in patients with low anterior resection syndrome.

Rectal cancer accounts for approximately one-third of cases of colorectal cancer, with its incidence worldwide predicted to increase to 2.5 million new cases in 2035 (17). Rectal cancer patients are more willing to accept low anterior resection of the rectum with total mesorectal excision in order to avoid permanent colostomy, which is associated with abdominoperineal resection (18). However, LARS may be a potential complication of this surgery. During SSOs for rectal cancer, it is important to preserve the pelvic nerve, which has been considered a core factor in avoiding LARS. In addition, in terms of pelvic nerve preservation, laparoscopic surgery is a good treatment option with a significant advantage due to the visualization of pelvic nerve preservation (19). In this study, more than 50% of patients had undergone laparoscopic surgery. Fecal incontinence is one of the most common symptoms of LARS (20). Kim KH et al. (21) evaluated the functional outcomes of biofeedback treatment for rectal cancer patients with fecal incontinence after surgery and found significant improvements in fecal incontinence score, number of bowel movements, and anorectal manometry data in patients who received biofeedback therapy. Meanwhile, Liang Z et al. (22) retrospectively reviewed data from 61 patients with LARS, and their results showed that biofeedback therapy is effective in improving the symptoms of fecal incontinence and anal pressure among rectal cancer patients after restorative resection. However, Liang Z et al. (22) reported that the efficacy of PFMEs for LARS is approximately 70%, with patients’ symptoms being only partially improved, and the factors influencing the functional outcomes of PFMEs for LARS patients are poorly understood. The authors found that current smoking status, number of biofeedback therapy cycles, and the use of laparoscopic surgery were factors influencing the effectiveness of PFMEs. Notably, these two studies employed retrospective designs. The latest study (23) found that the LARS scores, ColoRectal Functioning Outcome scores, and frequency of bowel movements of patients who underwent pelvic floor muscle training were significantly lower than those of a control group, which is a similar finding to that of our study.

The current prospective clinical trial indicated that an intervention consisting of pelvic floor muscle exercises improved maximal resting pressure and maximal squeeze pressure in LARS patients compared with a control intervention. Furthermore, LARS patients who received the PFME intervention reported significant improvements in stool frequency, symptoms of fecal incontinence (Wexner score), and stool consistency compared with those in the control condition. Allgayer H et al. (24) reported that irradiated patients who received pelvic floor exercise/biofeedback interventions showed short- and long-term training effects on incontinence score, and these results are in agreement with those of our study. Our study also assessed patients’ mental health; the outcomes showed that LARS patients experienced anxiety and depression, both of which could be significantly relieved after 4 weeks of PFMEs.

Some limitations of the study should be noted. A major limitation was that incontinence scores were subjectively recorded through patients’ self-reports. Although parameters of anorectal manometry may be considered objective data, patients’ experiences were also important, and this may have altered the outcomes. In addition, patients who could not complete the pelvic floor exercises were excluded, and the outcomes of this study were analyzed using per-protocol analysis instead of intention-to-treat analysis, which may affect the authenticity of the findings. Moreover, the sample size was small, and therefore large-scale multicenter studies need to be conducted in the future.

## Conclusions

Pelvic floor muscle exercises might be an effective treatment and could alleviate symptoms and improve rectal function and mental health in patients with low anterior resection syndrome.

## Data availability statement

The original contributions presented in the study are included in the article/supplementary material. Further inquiries can be directed to the corresponding author.

## Ethics statement

The protocol was approved by the Third Affiliated Hospital of Anhui Medical University (No. 2022-75). The patients/participants provided their written informed consent to participate in this study.

## Author contributions

WY contributed to the design of the study. WY, HS, HL, and DD conducted the study and collected the data. WY interpreted the data and drafted the manuscript. WY contributed to revision of the manuscript and provided critical opinions. All authors contributed to the article and approved the submitted version.
